# A Novel Energy-Efficient Approach for Human Activity Recognition

**DOI:** 10.3390/s17092064

**Published:** 2017-09-08

**Authors:** Lingxiang Zheng, Dihong Wu, Xiaoyang Ruan, Shaolin Weng, Ao Peng, Biyu Tang, Hai Lu, Haibin Shi, Huiru Zheng

**Affiliations:** 1 School of Information Science and Engineering, Xiamen University, Xiamen 361005, China; xmuwudh@stu.xmu.edu.cn (D.W.); ruanxiaoyang@stu.xmu.edu.cn (X.R.); 23320141153268@stu.xmu.edu.cn (S.W.); pa@xmu.edu.cn (A.P.); tby@xmu.edu.cn (B.T.); luhai@xmu.edu.cn (H.L.); shihaibin@xmu.edu.cn (H.S.); 2 School of Computing, Ulster University, Newtownabbey, CO Antrim BT37 0QB, UK; h.zheng@ulster.ac.uk

**Keywords:** activity recognition, low power consumption, low sampling rate, energy-efficient classifier

## Abstract

In this paper, we propose a novel energy-efficient approach for mobile activity recognition system (ARS) to detect human activities. The proposed energy-efficient ARS, using low sampling rates, can achieve high recognition accuracy and low energy consumption. A novel classifier that integrates hierarchical support vector machine and context-based classification (HSVMCC) is presented to achieve a high accuracy of activity recognition when the sampling rate is less than the activity frequency, i.e., the Nyquist sampling theorem is not satisfied. We tested the proposed energy-efficient approach with the data collected from 20 volunteers (14 males and six females) and the average recognition accuracy of around 96.0% was achieved. Results show that using a low sampling rate of 1Hz can save 17.3% and 59.6% of energy compared with the sampling rates of 5 Hz and 50 Hz. The proposed low sampling rate approach can greatly reduce the power consumption while maintaining high activity recognition accuracy. The composition of power consumption in online ARS is also investigated in this paper.

## 1. Introduction

Human activity recognition plays a crucial role in pervasive computing. Many applications for healthcare, sports, security agencies and context-aware services applications have emerged [1,2]. For example, life logs collected by smart mobile phone sensors (such as accelerometers) have been used to provide personalized health care [3]. Vermeulen et al. [4] developed a smartphone-based falls detection application to help elderly people. Zhou et al. [5] implemented a phone system for indoor pedestrian localization. Google Now is one of the emerging smart applications that provide context-aware services. It calculates and pushes relevant information automatically to mobile users based on their current locations [6].

The history of human activity recognition can be traced back to the late 1990s [7]. Four sensors (accelerometers) were placed on different positions of body to detect human activities (lying, sitting, sitting/talking, sitting/operating, standing, walking, upstairs, downstairs, and cycling). Randle and Muller [8] used a single wired biaxial accelerometer to classify six activities (sitting, standing, walking, running, upstairs, and downstairs) in 2000. However, the early systems are not easy to use.

Thanks to the development of microelectronics and computer systems, the sensors and mobile devices are now with higher computational capability, smaller size and more acceptable usability. The studies on activity recognition systems (ARS), especially smartphone based activity recognition system (ARS), have been set off a booming in recent years [9,10,11,12,13]. The accelerometers and gyroscopes embedded in smart phones have been used to collect raw activity data in ARS. Smart phones have become one of the most indispensable parts of life when comparing with other special devices [13,14]. It is now a relative low-cost device for both developers and users.

The smart phone based ARS can be divided into two types. One is online activity recognition systems, i.e., data collection, data processing and classification are carried out locally on the mobile phones [11,15]. The other is offline activity recognition systems, i.e., the classification is carried out non-real-time, or offline. Similar to other research [16], we consider an ARS in which the classification is carried out in a remote server or cloud as an offline ARS because the classification becomes non-real-time when the phone has no internet connection.

An online ARS can recognize the user’s behavior and provide the feedback in real time to support user’s daily life [17]. A number of studies on online ARS have been carried out. Anjum et al. [18] developed an application for recognizing a number of activities, including driving and cycling, with an average accuracy of greater than 95%. Kose et al. [15] investigated the performance of different classifiers and used accelerometer of the smartphone to classify four activities (sitting, standing, walking, and running). Schindhelm et al. [19] explored the capability of using smartphone (HTC hero) sensors for the detection of steps and movement/activity types. Martín et al. [20] presented the work of using smartphone for activity recognition without interfering user’s life.

Although the previous research work has achieved good accuracy in activity detection, there are few reports on power consumption. The power consumption is one of the main challenges [11], especially for the online ARS. The mobile phones are usually used for making phone calls or Internet surfing, so the power consumption of the online ARS must be reduced as low as possible. In order to solve this issue, one straightforward method is to reduce the number of sensors—for example, turning off the Global Positioning System (GPS) while the user is indoors or applying some energy-efficient sensors (such as accelerometers, gyroscopes) [10,21,22] instead. The other approach is to lower the sampling rate. However, in most studies [13], the sampling rates were still high because they followed the Nyquist theorem, i.e., the sampling rate must be equal to or higher than twice the signal frequency so that no actual information will be lost during the sampling process.

In this paper, we propose an energy-efficient ARS that uses low sampling rate and can still achieve high accuracy. A theoretically proof of the rationale of using low sampling rate in ARS is presented. A novel classifier is also proposed and developed to improve the performance of activity recognition. The proposed system consists of three components: (a) sensors using the proposed low sampling rate for data collection; (b) feature extraction for training and classification; (c) the proposed classifier which integrates hierarchical support vector machine (H-SVM) with context-based classification (HSVMCC) to detect user’s activities.

The rest of the paper is organized as follows. In Section 2, we briefly describe the related work. Section 3 details the proposed energy-efficient system, including data collection using low sampling rate, feature extraction method, the proposed HSVMCC algorithm, and the composition of power consumption in online ARS. The discussion of experiments and results is presented in Section 4. The paper is concluded by the summary of merits, limitations and future work in Section 5.

## 2. Related Work

The common approach of saving energy consumption in online ARS is to detect the mobile status and user’s location or activities and then turn on/off some unused sensors [22,23,24]. Wang et al. [24] presented a novel design framework for an Energy Efficient Mobile Sensing System (EEMSS). Using a hierarchical sensor management strategy to recognize user states and detect state transitions, the EEMSS significantly improved battery life of the device.

Adopting one tri-axial accelerometer, Lius et al. [13] detected activities (including walking, jumping, immobile, running, up, down, cycling and driving) using the sampling rate varying from 32 Hz to 50 Hz. An average accuracy of 98% was achieved. Discrete variables were used to reduce the calculation costs and save the energy. However, the sampling rate is still too high to maximize energy savings.

Reddy et al. [25] employed the GPS and accelerometer to detect activities (including stationary, walking, running, biking or motorized transport). Although the sampling rate was set to 1 Hz only, the GPS is not an energy-efficient sensor and cannot be used indoors.

Similarly, aiming to detect human mobility states, Oshin et al. [10] used an accelerometer at a sampling rate of 4 Hz. The results showed an overall average accuracy of 92%. However, it failed to detect some regular indoor activities, such as climbing stairs, in contrast to other studies [12,18,26]. The rationale of using the sampling rate of 4 Hz was not presented in the paper.

Applying a tri-accelerometer at the sampling rate of 2 Hz, Liang et al. [27] managed to obtain the average accuracy of 89% detecting human activities (standing, sitting, lying, walking, running, jumping, ascending, descending, cycling and driving). However, no justification was provided for the choice of sampling rate. It also lacked the accurate evaluation of power performance.

Activity recognition plays an important role in the area of pervasive healthcare. Liang et al. [28] proposed a hierarchical method to recognize user activities based on a single tri-axial accelerometer in smart phones for health monitoring. 

Li et al. [29] proposed to leverage machine learning technologies for improving the energy efficiency of multiple high-energy-consuming context sensors by trading off the sensing accuracy. 

For the purpose of utilizing available energy efficiently while achieving a desired activity recognition accuracy, Zappi et al. [30] investigated the benefits of dynamic sensor selection. It introduced and characterized an activity recognition method with the help of an underlying run-time sensor selection scheme.

Mortazavi et al. [31] presented a multiple model approach to classifying movements in exergame environment with fine-grain motions. Expert knowledge was applied to identify similar movements. Each submodel was modeled using a one to many support vector machine (SVM) with nonlinear kernel. Although the multiple model approach achieved a good classification performance, the study didn’t consider the power consumption either in algorithms or in data sampling, where a sampling rate of 50 Hz was used.

In our previous work [32], we have experimentally tested and confirmed that the sampling rate of 1 Hz could achieve high performance for detecting activities (including sitting, standing, walking, and running) in an offline ARS.

Considering the above-related work, we have carried out a theoretical analysis on why using a low sampling rate in ARS can also achieve high performance. Experiments based on the smartphone have also been undertaken to evaluate the power consumption of the online ARS with different sampling rates. Furthermore, the recognition of climbing stairs activities (upstairs and downstairs) is also included in the proposed ARS.

## 3. Energy-Efficient Activity Recognition System

**Figure 1:**
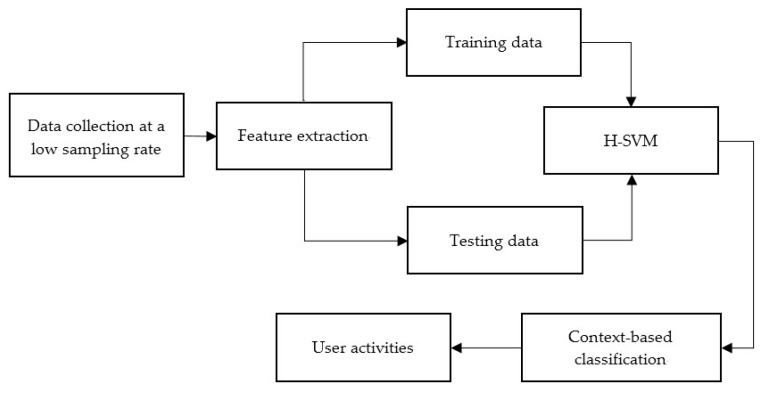
System architecture.

The aim of this research is to build a user-independent and energy-efficient online ARS with high accuracy. The proposed system, as shown in Figure 1, includes data collection, feature extraction, and the training and classification (H-SVM and context-based classification). The system does not contain data processing before the feature extraction because the data obtained have been preprocessed by the phone’s built-in filters.

### 3.1. Data Collection at a Low Sampling Rate

The types of sensors and the sampling rates are two factors that must be considered during data collection in ARS. The barometer, accelerometer and gyroscope are the sensors usually used in ARS.

Barometer is the sensor for measuring altitude or height. Using low sampling rate of barometer can detect whether the user is climbing stairs or not.

Inertial measurement units (IMUs), such as accelerometer and gyroscope, are used to measure the user motion. In previous studies [1], the sampling rate of these sensors (such as accelerometers) in ARS was set between 10 Hz and 100 Hz. It is a general view that a high sampling rate can avoid the information loss of signals. Some research also claimed that high sampling rate could achieve high accuracy of recognition [33]. However, the higher the sampling rate is, the more energy consumed. The trade-off between the sampling rate and the power consumption has become one important concern in most energy-efficient ARS.

In our research, we proposed a solution to solve the contradiction between the sampling rate and the power consumption, that is, using a low sampling rate of IMU in ARS to achieve a similar recognition accuracy, compared with using high sampling rates.

For human activity recognition, the purpose of sampling is not to restore the raw signals of activities, but to detect different activities according to the statistical properties of signals, such as means, variance, and maximum. It is considered that using high sampling rate can capture all the details of the person’s movements, and this would benefit the recognition of activity [34]. However, the signal information would not be lost if the sampling rate agreed with the Nyquist theorem, which means that the statistical properties of using low or high sampling rate are consistent. When the sampling rate is less than the frequency required by the Nyquist theorem, we suggested that adding more sampling periods can acquire the consistent statistical properties, which is demonstrated as follows:

Set the frequency of activity as $F_{a}$, the sampling rate as $F_{S}$, and the sampling period as T.

For different sampling rates of $F_{S_{1}}$ and $F_{S_{2}}$, if they agree with the following conditions:

(1) $$F_{S_{1}}\%F_{S_{2}}\neq 0$$

There exists Equation (2):

(2) $$F_{S_{1}}\times T_{1}=F_{S_{2}}\times T_{2}\;\;T_{1}\in \left\{1,2,\ldots ,n\right\},F_{S_{1}}\times T_{1}\in \left\{1,2,\ldots ,n\right\}$$

where $T_{1}$ and $T_{2}$ are sampling periods.

The elements of dataset $X_{1}=\left\{x_{1},x_{2},\ldots ,x_{n}\right\}$ obtained at the sampling rate of $F_{S_{1}}$ and sampling period of $T_{1}$ are the same with dataset $Y_{1}=\left\{y_{1},y,\ldots ,y_{n}\right\}$ obtained at the sampling rate of $F_{S_{2}}$ and sampling period of $T_{2}$. Thus, the statistical properties of the dataset $X_{1}$ and the dataset $Y_{1}$ are the same, if the sampling period is long enough.

If sampling rate $F_{S_{3}}$ is less than the frequency required by the Nyquist theorem $F_{a}$, which is:

(3) $$F_{S_{3}}<2F_{a}$$

Human activity signal is a non-strict period, so $F_{a}$ is not a determined value, but a fluctuating value. The relation between $2F_{a}$ and $F_{S_{3}}$ satisfied the Equation (1). The same as above, the elements of dataset $D=\left\{d_{1},d_{2},\ldots ,d_{n}\right\}$ obtained at the sampling rate of $2F_{a}$ and the sampling period of T has the same statistical properties as the dataset $D^{\prime }=\left\{d_{1}^{\prime },d_{2}^{\prime },\ldots ,d_{n}^{\prime }\right\}$ obtained at the sampling rate of $F_{S_{3}}$ and the sampling period of $T_{3}$.

Combined with the formulas above, time period $T_{3}$ is calculated as Equation (4):

(4) T3=(2Fa×T)FS3

Therefore, when we use a low sampling rate that does not agree with the Nyquist theorem in ARS, we can add the sampling time to ensure the same statistical properties.

### 3.2. Hierarchical Support Vector Machine (H-SVM)

Support vector machine (SVM) is a supervised learning algorithm. The basic SVM model is the probability of a binary classification. In order to deal with multiple classes, Liu et al. [35] proposed an adaptive hierarchical multi-class SVM classification scheme at the training stage.

In this paper, the *k*-means clustering algorithm was used in training the H-SVM classifier.

The training algorithm is summarized in Algorithm 1.
**Algorithm 1** Training algorithm1: Construct a feature set ($\left\{f_{1},f_{2},\ldots ,f_{n},\left(f_{i_{1},}f_{j_{1}}\right),\left(f_{i_{2},}f_{j_{2}}\right),\ldots ,\left(f_{1},f_{i},\ldots ,f_{m}\right)\right\}$). The feature set is the combination of all features.2: Sorting features according to the power consumption of the sensor and the computational cost of feature extraction. The lower power consumption of the sensor ranks the higher. Features with lower computational cost have in higher priority when the sensor is the same.3: Selecting *m* top features with the higher priority from the feature set.4: For each of these *m* features, set the whole dataset as DataSets_In.5: Input DataSets_In. do6: {7:  Set parameter of clustering *k* = 28:  Use *k*-means clustering algorithm to get two cluster A and B. The two clusters are the subsets of DataSets_In. Evaluate the performance of *k*-means clustering, select the optimal ones according the accuracy and the equilibrium of the two subset.9:  Input subsets A and B10:  Training a binary classifier SVM11:  SVM_*n* = SVM (*n* = 1, 2, 3……)12:  DataSets_In = subsets A or B respectively13: } while DataSets_In only contains data from a single class14: The resulting classifier (i.e., final classifier) is a one-node SVM classifier, as an *N*-class classification needs an *N-1* node classifier.

### 3.3. Context-Based Classification

During the study, we found that there are two reasons that some errors may occur in the activity recognition: (1) features were similar between two activities; (2) some measurement errors. To correct these errors, we proposed a context-based classification approach. Context-based classification is a method that combining the previous variables’ information and the following variables’ information during the analysis of the current variable. It can effectively eliminate the individual errors.

**Figure 2:**
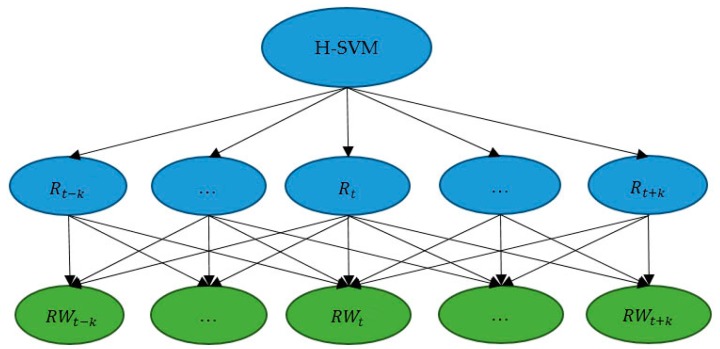
The context-based classification.

Human activities are continuous processes. Therefore, the previous recognition results and the following recognition results can be used to check and correct the current recognition result. The process is archived by using a sliding window (the window length is *2k + 1*) to correct the result at time *t*, as shown in Figure 2.

In Figure 2, the variable $R_{t}$ is the result of the H-SVM model at time t, and $RW_{t}$ is the corrected result after context-based classification. $RW_{t}$ is defined as the mode (the most frequent result) among $\left\{R_{t-k},R_{t-k+1},\ldots ,R_{t+k}\right\}$. We assume that the probability of recognition errors ψ is independent identically distributed, the accuracy at time *t* ($Accuracy_{t}$) with values of *k* is showed as follows:

(5) $$Accuracy_{t}={\left(1-\psi \right)}^{2k+1}+C_{2k+1}^{1}{\left(1-\psi \right)}^{2k}\psi +\ldots +C_{2k+1}^{2k}\left(1-\psi \right)\psi^{2k}+\psi^{2k+1}$$

The algorithm is summarized in Algorithm 2:
**Algorithm 2** Context-based classification Algorithm1: Initialize the data buffer $\left\{Result_{1},Result_{2},\ldots ,Result_{2k+1}\right\}$2: while R is input from H-SVM do3: {4:  If *n* < 2*k* + 2 then5:   $Result_{n}=R\left(n=1,2,\ldots ,2k+1\right)$6:   $RW_{T}=Rt$7:  else8:   $\{Mode_{1},Mode_{2},\ldots \}=MajorityOf\left\{Result_{1},Result_{2},\ldots ,Result_{2k+1}\right\}$9:     if $Result_{k+1}\in \left\{\left\{Mode_{1}\right\},\left\{Mode_{2}\right\},\ldots \right\}$ then10:       $RW_{t}=Result_{k+1}=R_{t}$11:     else12:       $RW_{t}=Mode_{1}$13: }14: end while

Firstly, variables $\left\{Result_{1},Result_{2},\ldots ,Result_{2k+1}\right\}$ are initilised as data buffer. Then, for each R received, it is placed to the data buffer $\left\{Result_{1},Result_{2},\ldots ,Result_{2k+1}\right\}$. For the first R (which equals to $R_{t-k}$) input from H-SVM, the $RW_{t}$ is set as $R_{t}$. For the following R, $RW_{t}$ does not change. When the 2*k* + 1 of R are all input, the majority $\left\{Mode_{1},Mode_{2},\ldots \right\}$ of the data buffer is calculated. If $Result_{k+1}$ (here, $Result_{k+1}$ equals to $R_{t}$) belongs to $\left\{Mode_{1},Mode_{2},\ldots \right\}$, and the $RW_{t}$ is set as $Result_{k+1}$; otherwise, the $RW_{t}$ is set as $Mode_{1}$. Then, the data buffer is shifted right by one to store the next $R_{t}$. For the next RW, the steps of calculating RW are the same as $RW_{t}$. The algorithm is stopped when R is no longer received.

Time delay is the main defect of the context-based classification algorithm. It is equal to the value of *k*. It increases with the growth of the sliding window size. Thus, it is important to choose a suitable sliding time window size (that is, the values of *2k + 1*) in an online ARS.

## 4. Experiments and Discussion

The experiments include four parts: (1) data collection; (2) parameter selections; (3) classification performance; and (4) the power consumption of the proposed energy-efficient online ARS.

### 4.1. Data Collection

**Figure 3:**
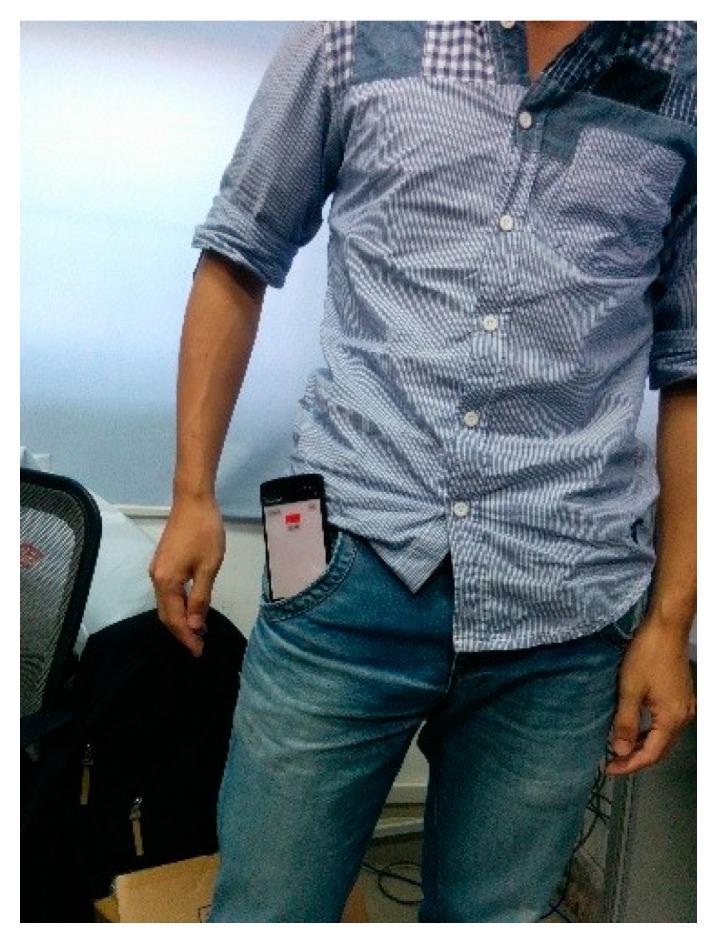
Illustration of the placement of the phone on the participant’s body.

For data collection, a smartphone (Nexus 5, Google Inc., Mountain View, CA, United States of America) was placed in the right-front pocket of the pants, showed in Figure 3. The sensors used in the experiments were barometer and accelerometer inside the smartphone. As shown in Table 1, four independent data collections were carried out separately in the study.

Data collection 1 (Training datasets): the sampling rate was set to 1 Hz, the time window was 5 s, without overlap. One volunteer (male, 23 years old, healthy student) was asked to perform six types of activities: sitting, standing, walking, running, climbing upstairs and going downstairs. The volunteer sat and stood indoors, walked in the corridor or in the room, ran on a treadmill at 9 km/h, climbed upstairs and went downstairs in our lab building, which is six-floors. Each activity was carried out 15 times (15 samples collected). In total, 90 samples were collected as the training data-sets.

Data collection 2: The sampling rate was set to 1 Hz, the time window was 5 s, without overlap. Twenty volunteers (14 males and six females, ages between 22 to 25) participated in the data collection. The volunteers were asked to undertake the six activities as described in the Data collection 1. Each activity (sitting, standing, walking and running) was consecutively carried out for 5 min. The volunteers were asked to climb upstairs from the second floor to the sixth floor and go downstairs from the sixth floor to the second floor, five times repeatedly. All these data collected in Data collection 2 are only used for testing, as shown in Table 2. For each activity, we removed the data of the first time window (5 s) and the last time window (5 s) to ensure that the data obtained only contained one type of activity.

To compare the accuracy of activity recognition at different sampling rates, five volunteers (from the above 20 volunteers in the Data collection 2) participated again in the following two new data collections.

Data collection 3: The purpose of this data collection is to verify that using a low sampling rate (1 Hz) can also achieve high accuracy of recognition, compared with the sampling rate agreed with the Nyquist theorem. Thus, the sampling rate was set as 5 Hz, which agreed with the Nyquist theorem and was close to twice the frequency of human activity obtained by phone sensor. The time window was 1 s.

Data collection 4: The aim of this data collection is to verify that different sampling rates that agreed with the Nyquist theorem achieve almost the same accuracy. Activity data were collected at the sampling rates of 10 Hz and then 50 Hz. The time window of different sampling rates was 1 s.

### 4.2. The K-Means Clustering and H-SVM

In general, the feature extraction aims to identify the main characteristics that accurately represented the original data [36]. The process is to find the most useful, valid and meaningful information to recognize activities with high accuracy. In previous studies [1,10,13], the common features include time domains and frequency domains, such as means, standard deviation, magnitude of acceleration and FFT (Fast Fourier Transform). There are no fixed features that are suitable for all ARS.

In this paper, we firstly constructed a feature set. The feature set is the combination of all features, that is $P_{d}$ (pressure difference), $P_{d_{abs}}$ (absolute value of pressure difference), $X_{means},Y_{means},Z_{means}$ (the means of *X*-/*Y*-/*Z*-axis accelerometer values), and $T_{waves}$(the sum of root mean squares of the difference of adjacent points in a time window).

After constructing the feature set, algorithm 1 was applied to feature selections and classification. Based on the power consumption of the sensor and the computational cost of feature extraction, *m* features ($P_{d},P_{d_{abs}},Y_{means},T_{waves}$) with the higher priority from the set of optimal features were selected. 

(1) Pressure difference Pd: The difference of pressure value is measured by barometer built-in the mobile phone, as shown in Equation (6). The barometer value is considered as height changing. When the altitude increases, the pressure value decreases, and vice versa:

(6) $$P_{d}=p_{n}-p_{0}$$

where $p_{n}$ is the last pressure value and $p_{0}$ is the first pressure value in the time window (sampling period). The $P_{d}$ value is negative when the user climbs upstairs, and it is positive when going downstairs.

(2) The absolute value of pressure difference ($P_{d_{abs}}$): The $P_{d_{abs}}$ is calculated as follows:

(7) $$P_{d_{abs}}=abs\left(P_{d}\right)$$

(3) *X*-/*Y*-/*Z*-axis accelerometer value ($X_{means},Y_{means},Z_{means}$): This is the means of the *X*-/*Y*-/*Z*-axis accelerometer values. The values of tri-axial accelerometer we got from the smartphone (Android API) contained the gravity values. The following is the calculation for $X_{means},Y_{means},Z_{means}$:

(8) Xmeans=x1+x2+x3nYmeans=y1+y2+y3nZmeans=z1+z2+z3n

(4) The wave of three-axis accelerometer ($T_{waves}$): this is the sum of the RMS (Root Mean Square) of the difference of adjacent points in a time window, and can be calculated using Equation (9):

(9) $$T_{wave}=\sum_{i=0}^{n}\sqrt{{\left(AccX_{i+1}-AccX_{i}\right)}^{{}^{2}}+{\left(AccY_{i+1}-AccY_{i}\right)}^{{}^{2}}+{\left(AccZ_{i+1}-AccZ_{i}\right)}^{{}^{2}}}$$

where $AccX_{i},AccY_{i},AccZ_{i}$ are the three-axis values of accelerometer at time stamp *i*, respectively.

**Figure 4:**
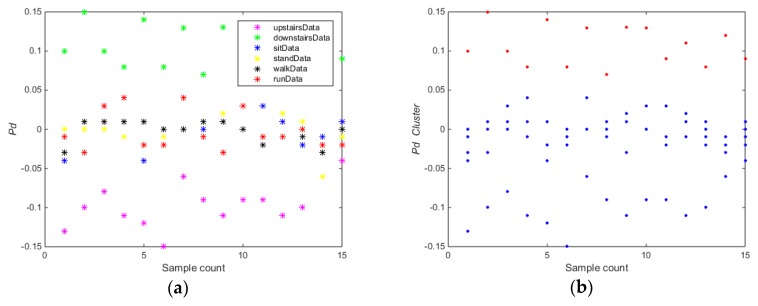

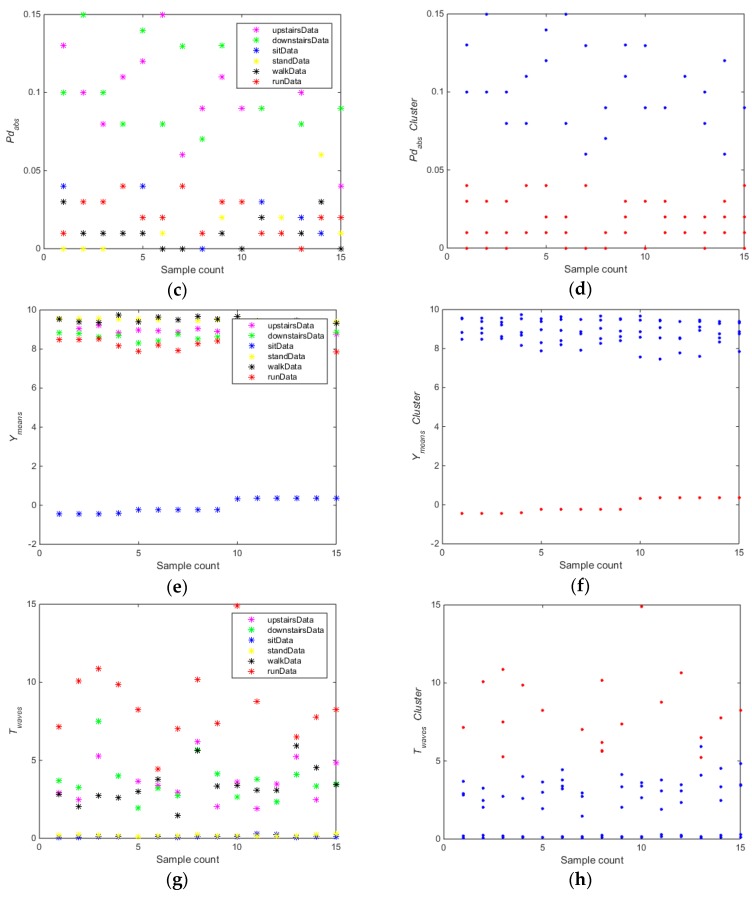
(**a**) $P_{d}$ value; (**b**) results of *k*-means clustering of $P_{d}$ feature; (**c**) $P_{d_{abs}}$ value; (**d**) results of *k*-means clustering of $P_{d_{abs}}$ feature; (**e**) $Y_{means}$ value; (**f**) results of *k*-means clustering of $Y_{means}$ feature; (**g**) $T_{waves}$ value; (**h**) results of *k*-means clustering of $T_{waves}$ feature.

The training carried out on the whole dataset (Data collection 1) using algorithm 1. As shown in Figure 4, for the feature $P_{d}$ (Figure 4a,b), the whole training dataset was divided into subset A (downstairs) and B (upstairs, sitting, standing, walking, running). For the feature $P_{d_{abs}}$ (Figure 4c,d), the whole training dataset was divided into two subsets A (upstairs and downstairs) and subset B (sitting, standing, walking, running) using the *k*-means clustering algorithm. For the feature $Y_{means}$ (Figure 4e,f), the whole training dataset was divided into subset A (sitting) and B (downstairs, upstairs, standing, walking, running). For the feature $T_{waves}$ (Figure 4g,h), the whole training dataset was divided into subset A (running) and B (downstairs, upstairs, sitting, standing, walking).

As shown in Figure 4, the accuracy and partition degree of *k*-means clustering of different features were assessed. The equilibrium of two subsets was also considered. Figure 4b,d,f shows the results that *k*-means clustering can get good performance using the selected features, but only the feature $P_{d_{abs}}$ can meet the equilibrium requirement. Thus, the selected feature $P_{d_{abs}}$ is the optimal feature for training the first SVM classifier (SVM1). In the Algorithm 1, the data were randomly divided into 80% for training and 20% for testing in order to select the optimal features and build the SVM classification models.

According to Algorithm 2, for subset (upstairs or downstairs), feature $P_{d}$ is the optimal feature for training SVM classifier (SVM2) to partition the upstairs or downstairs because it is the most accuracy ones. For subset (sitting, standing, walking, running), feature $Y_{means}$ is suitable for classification (SVM3), dividing dataset (sitting, standing, walking, running) into subset (sitting) and subset (standing, walking, running) with the highest accuracy. In addition, $Y_{means}$ is also the optimal feature for dividing dataset (standing, walking, running) into subset (standing, walking) and running (SVM4). Finally, the $T_{waves}$ is used for partition standing and walking (SVM5). The whole H-SVM classification model is shown as follows.

**Figure 5:**
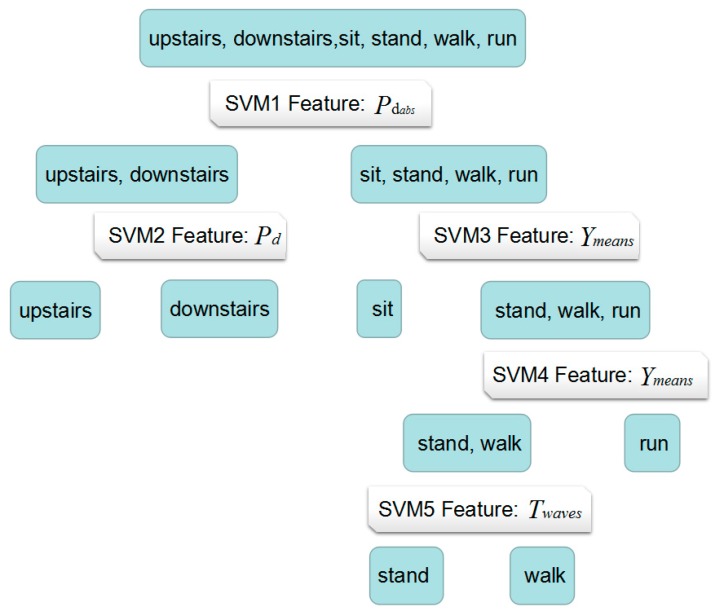
The whole H-SVM classification model.

The whole training dataset (Data collection 1) contains 90 samples. After training, the five-node SVM classifier was built. As illustrated in Figure 5, the dataset is divided into two sets whether the activity is climbing stairs or not. If the classification result of classifier SVM1 is climbing stairs, classifier SVM2 is used to judge if the activity is climbing upstairs or going downstairs. If the result of SVM1 is not stair climbing, classifier SVM3 is applied to classify sitting or standing, walking, running and then classifier SVM4 will contribute to recognize standing, walking or running. Finally, classifier SVM5 is used to differentiate standing or walking. Considering the *k*-means clustering results discussed before, $P_{d_{abs}}$ can be used as the input feature for SVM1 to detect climbing stairs or not. Furthermore, $P_{d}$, $T_{waves}$ can be used as input feature for SVM2 and SVM5, respectively, and $Y_{means}$ can be input as feature for SVM3 and SVM4. These may reflect the body movement efforts and acceleration patterns when carrying out different types of activities, that is: (1) the pressure used in climbing stairs activities is different from activities on flat ground; (2) changing of acceleration on the *Y*-axis can be used to differentiate the sitting status from standing/walking/running and further differentiate running from walking/standing; (3) information of changes in all three directions needs to be taken into account in order to classify standing from walking.

### 4.3. The Parameter Settings of Proposed ARS.

The sampling rate and time window of accelerometer during data collection and sliding window size of context-based classification are three crucial parameters that may affect the power consumption and accuracy of proposed ARS.

The frequency of human activity is about 2 Hz. For example, the frequency of going downstairs with fast speed is less than 2 Hz, and the step time of fast walking is 0.35 s/step [37]. The time windows were usually 1 s in previous studies [38]. In our research, the sampling rate of accelerometer was 1 Hz. According to Equation (4), the time window was about 5 s.

**Figure 6:**
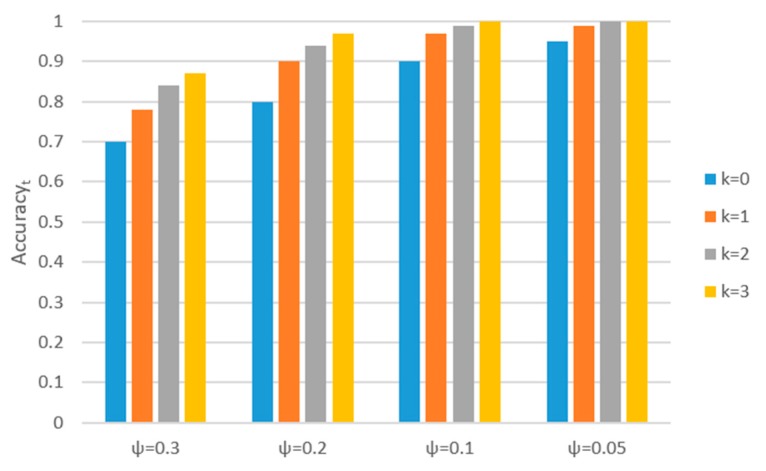
The $Accuracy_{t}$ of different sliding window length *k*.

In Section 3.3, we proposed context-based classification to improve the accuracy of recognition. For different values of the probability of recognition error ψ (0.3, 0.2, 0.1, and 0.05), the $Accuracy_{t}$ of different sliding window size (2*k* + 1) is shown in Figure 6:

Figure 6 shows that the $Accuracy_{t}$ has improved with the increase of *k*-values. For example, the $Accuracy_{t}$ has improved 8% with the change of value *k* from 0 to 1 when ψ = 0.3. However, the time delay will also increase, which may be harmful to the online ARS. Especially when the recognition error ψ is becoming closer to 0, with the increase of value *k*, the improvement of $Accuracy_{t}$ is becoming smaller, but the time delay is becoming greater.

The classification performance of our proposed ARS shows that the largest recognition error ψ is less than 0.2 and the average recognition error is less than 0.1. As shown in Figure 6, no matter ψ = 0.2 or ψ = 0.1 or ψ = 0.05, the accuracy is improved quickly when *k* is increased from 0 to 1. However, the improvement of $Accuracy_{t}$ slows down when *k* ≥ 1, but the time delay became greater. Therefore, the slide window size is set as 3 (*k* = 1). 

### 4.4. The Classification Performance of Proposed ARS

In this section, we assess the performance of proposed classifier and compare it with other classifiers. The classification accuracy of different sampling rates is also discussed.

#### 4.4.1. Performance of Different Classifiers

In our research, we used the H-SVM model and context-based classification. In order to analyze the performance of H-SVM, we used the training dataset (Data collection 1), testing dataset (Data collection 2) and features obtained from the mobile phone. The training and classification were carried out in Matlab 2014a (MathWorks Inc., Natick, MA, USA), using Libsvm library [39]. The training used the linear kernel, cost and without cross-validation. The features of H-SVM and the parameters of SVM used in Matlab were the same as those on the phone. The classification results are shown in Table 3.

**Figure 7:**
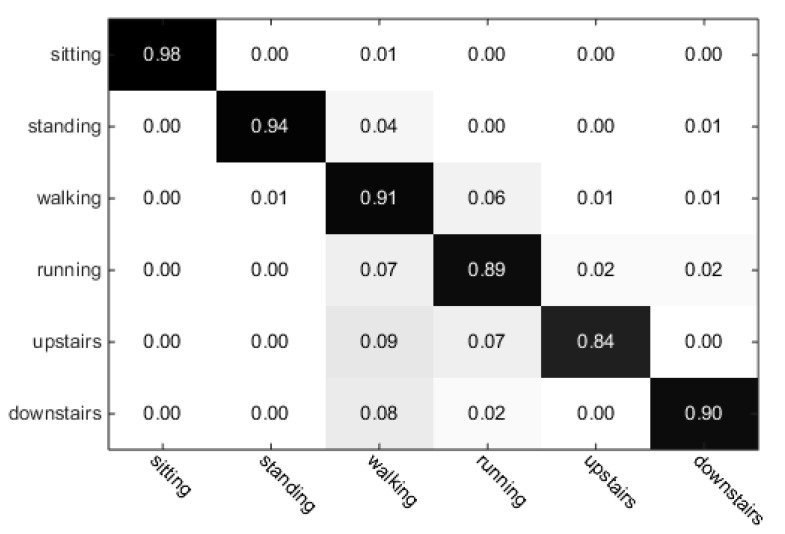
The classification results of climbing upstairs activity after H-SVM.

As shown in Table 3, the average accuracy of six activities is 90.9% and the weakest performance (the accuracy is only 83.8%) occurred when recognizing climbing upstairs. Upon close examination, we found that it was caused by the noise of the signals, which led to the misclassification in some discrete time windows. We randomly selected 200 continuous recognition results of climbing upstairs shown in Figure 7. In Figure 7, it can be seen that some activities of climbing upstairs were misclassified as other activities, such as standing, walking and running.

To reduce the impact of the noise and to improve the accuracy, we applied context-based classification after H-SVM. The results are shown in Table 4. The process of data collection, processing, training, and classification are all done by the phone. The accuracy values of six activities are increased by 1.8%, 3.1%, 5.5%, 4.7%, 8.3% and 6.6%, respectively, and the average accuracy of six activities is increased by 5.1%. The average accuracy of six activities of the proposed ARS is 96.0%, which is high enough for most applications.

**Figure 8:**
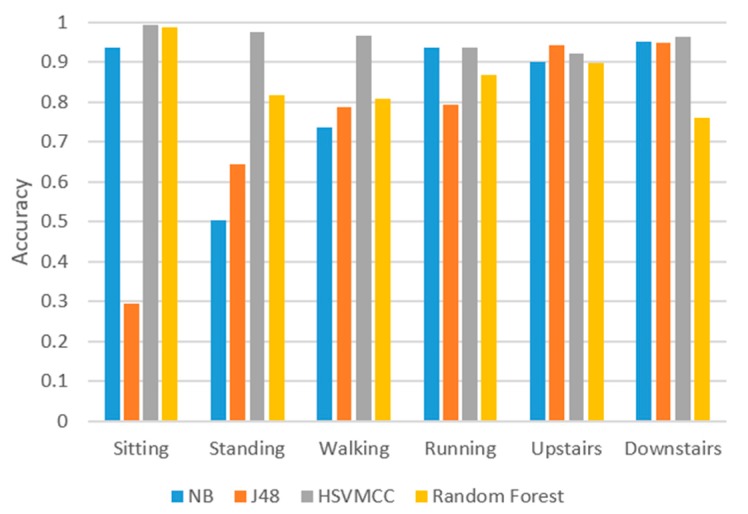
Comparison of the proposed ARS vs. classification models, J48, NB and Random Forest.

We compared our method with other classification algorithms such as J48 Naive Bayes (NB) and Random Forest (RF). The machine learning tool weka [40] was used in the study and the results are shown in Figure 8. We used the same training datasets (Data collection 1) described before to obtain the model of other classification algorithms. The universal parameters were selected for these classification algorithms. For J48, the parameter *C* was set as 0.25 and *M* was set as 2. For Random Forest, the parameter *I* was set as 100, *K* was set as 0 and *S* was set as 1. Then, we used all testing datasets (Data collection 2) in Table 2 to test the classifiers.

Figure 8 shows that the accuracies of the proposed method (HSVMCC) are more than 90% for all six activities. However, the accuracies of other algorithms vary between different activities. For sitting, the Random Forest (RF) achieves a high accuracy of 98.9%, while J48 obtained the lowest accuracy of 29.5%, but, for the ‘going downstairs’ activity, the accuracy of J48 is 94.8%, while the accuracy of Random Forest only achieves 76.1%.

**Figure 9:**
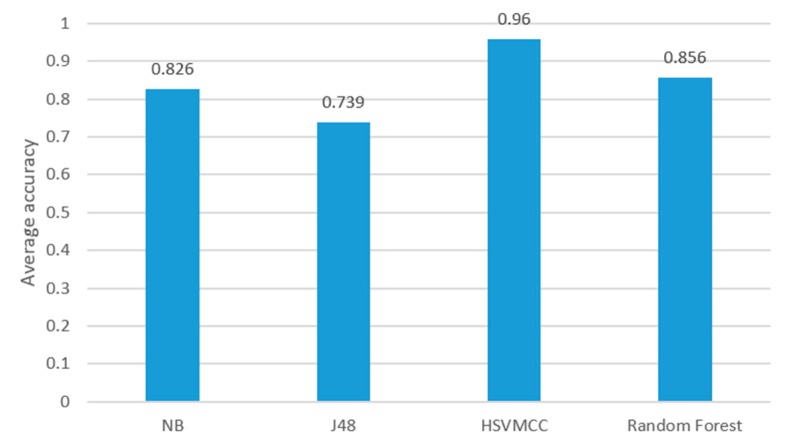
The comparison of average accuracy of the proposed AR system vs. other classifications.

Figure 9 shows the average accuracy of six activities of HSVMCC in comparison to Naive Bayes (NB), J48, and Random Forest (RF). The average classification accuracy for HSVNCC, NB, J48 and RF are 96%, 82.6%, 73.9%, and 85.6%, respectively. It can be concluded that the proposed HSVMCC outperformed other classifiers in terms of the average accuracy.

#### 4.4.2. The Accuracy of Different Sampling Rates

**Figure 10:**
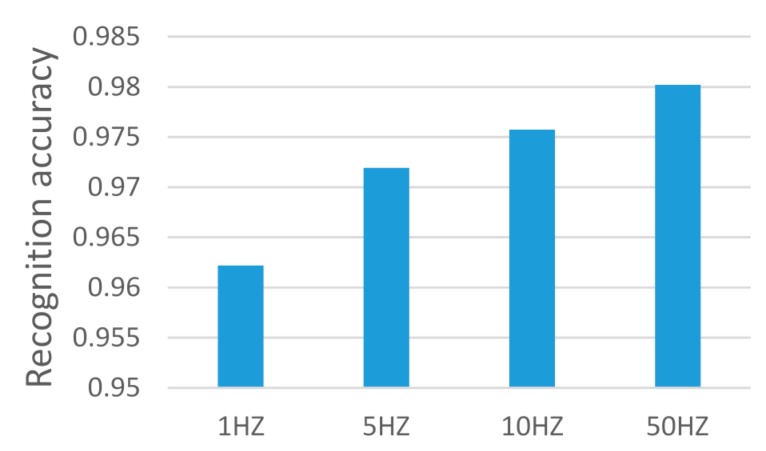
Recognition accuracy of activities at different sampling rates.

As mentioned before, we can use the sampling rate, which is less than the frequency required by the Nyquist theorem. Figure 10 shows the recognition results of ARS using the sampling rate of 1 Hz (less than the frequency required by the Nyquist theorem), 5 Hz (agreed with the Nyquist theorem), 10 Hz and 50 Hz. It can be observed that the accuracy of using 1 Hz sampling rate and using 5 Hz sampling rate are comparable, or similar. This means that, if the sampling rate is less than the frequency required by the Nyquist theorem, we can add the sampling period to achieve the similar accuracy of using the higher sampling rate that agrees with the Nyquist theorem.

Figure 10 also shows the accuracy has only improved slightly with the increase of the sampling rate from 1 Hz to 50 Hz, i.e., (1 Hz: 96.2%, 5 Hz: 97.2%, 10 Hz: 97.6%, 50 Hz: 98.0%). The accuracy of 1 Hz (96.2%) is sufficiently high for practical applications.

### 4.5. The Power Consumption of the Energy-Efficient ARS

The research about the compositions of energy consumption in ARS can help us to assess whether the proposed energy-efficient strategies are effective or not. Furthermore, the analysis of the composition of energy consumption in ARS can provide guidance for the researchers in energy-efficient fields.

An online ARS consists of data collection, data processing and activity recognition. Thus, the main composition of energy consumption in ARS can be divided into three parts. The first part is the power consumption used by the sensors. In our research, this part does not contain the data collection. The second part is the power consumption used in data processing, including data collection, feature extraction and data storage. The last part is the power consumption used by the activity recognition algorithm.

In our previous work [32], we proposed that the low sampling rate can decrease the power consumption. Power consumption for ARS is caused by the sensor running [10] or the total power consumption [13]. In this paper, we carried out the experiments to analyze the composition of power consumption in ARS.

We use the other mobile phone (Nexus 5, with an Android 4.4.2 system) for experiments. Firstly, we restored the phone to factory data to avoid power consumption caused by other applications, and we installed the requiring applications in the phone. Then, we put the phone in a shaker to do the experiments.

The experiments can be divided into two categories.

Category 1: we carried out the experiments in the shaker with the setting of 5 mm amplitude and 5–10 Hz variant-frequency vibration and a total of 17 experiments were undertaken.

Category 2: we carried out the experiments in the shaker with the static state and a total of 17 experiments were undertaken.

The purpose of contrasting two states (shaker and static state) is to simulate the real situations. We used the shaker to simulate the status of moving such as walking. Similarly, the static state was used to simulate standing and sitting status.

For each category, we conducted four experiments (sampling rate of 1 Hz, 5 Hz, 10 Hz, 50 Hz respectively) with the setting of running the whole ARS, four experiments with the setting of only running sensors, four experiments with the setting of running the ARS without activity recognition and result processing, four experiments with the setting of running the ARS without result processing and one experiment when the phone was on standby. The details are listed in Table 5.

**Figure 11:**
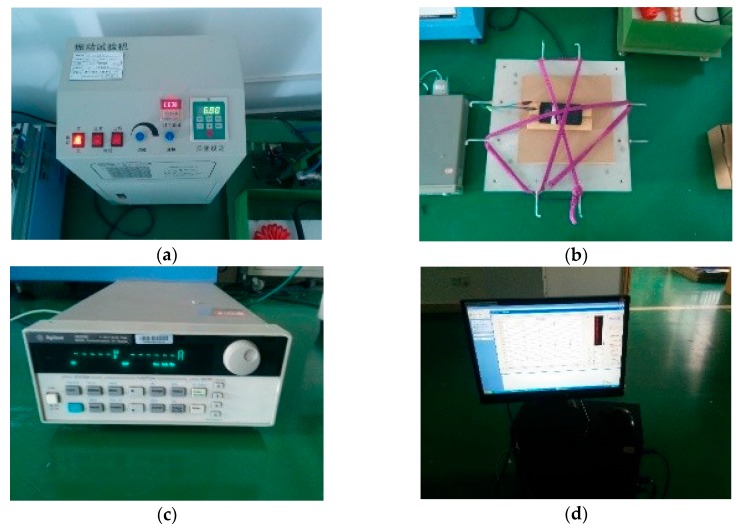
(**a**) Power test environments; (**b**) the phone was fixed in the shaker; (**c**) the external power; (**d**) computer used to control the experiments.

For each experiment, we fixed the mobile phone in the shaker (Figure 11a) and connected an external signal generator (3.8 V) to the mobile phone (Figure 11b), and then connected the signal generator with computer to collect data of current (the time is set as 20 min). We turned on the phone and started the application (five experiment settings shown in Table 5) under the experiment condition (shaker or static state). In the end, we clicked the button (“start to save data”) to collect the data of current.

**Figure 12:**
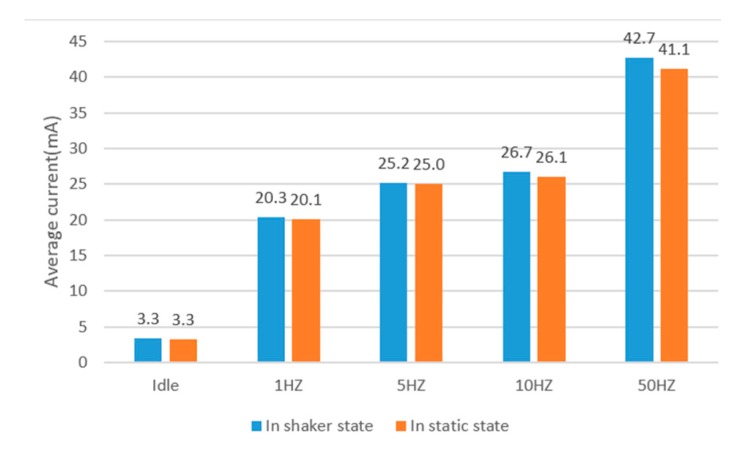
The average currents of the ARS at different sampling rates.

**Figure 13:**
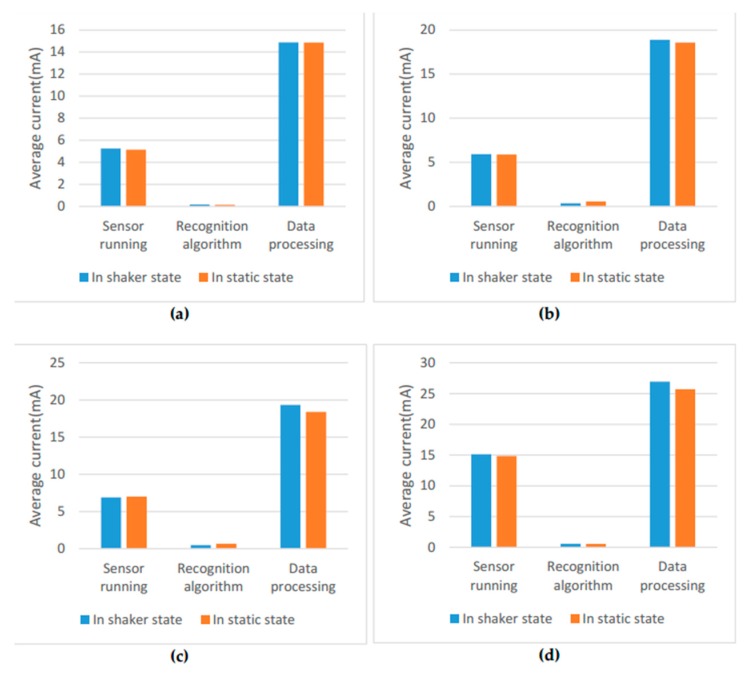
The average current in the sensor running, data processing and activity recognition. (**a**) ARS with a sampling rate of 1 Hz; (**b**) ARS with a sampling rate of 5 Hz; (**c**) ARS with a sampling rate of 10 Hz; (**d**) ARS with a sampling rate of 50 Hz.

Figure 12 illustrates the average current of ARS at different sampling rates. It shows that the average current increases with the increase of the sampling rate. The average current is 20.3 mA at 1 Hz, and it is 42.7 mA at 50 Hz when the phone is in the shaker state. The average current is 20.1 mA at 1 Hz, and it is 41.1 mA at 50 Hz when the phone is in the static state. It also infers that the power consumption of ARS at rate of 10 Hz has slightly increased compared with 5 Hz. There is a large increase of power consumption when the sampling rate changes from 10 Hz to 50 Hz. There are two main reasons. One reason is that the sensor running has a great increase when the rate changes from 10 Hz to 50 Hz (as shown in Figure 13). The other reason is the amount of data increase greatly when the sampling rate increases from 10 Hz to 50 Hz, which causes more power consumption in data processing.

Figure 13 also shows the average current of different parts in ARS. The data processing consumes most of the power in the online ARS. The second large power consumption is the sensor running. The power consumption of the proposed recognition algorithm is very small and can even be negligible. With the decrease of the sampling rate, the energy is saved in the sensor running and data processing for the reason that the amount of data is smaller.

We carried out another experiment to evaluate the power performance with different sampling rates. We turned on the phone when the phone was fully charged, and started phone application at four different sampling rates (1 Hz, 5 Hz, 10 Hz, 50 Hz) or idle state, respectively. Then, put the phone statically on the table, unplugged the charging cable and turned off the screen. After 24 h, we turned on the screen, stopped the application and recorded the data. Experiments on each sampling rate and the idle state were repeated four times. We also installed an external application called Battery Monitor Weight [41] on the phone to record the battery states (the recording interval was 2 min).

Another metric for evaluation Power Consumption Ratio (PCR) is introduced in this paper:

(10) PCR=PCfsPC50HZ

where $PC_{f_{s}}$ represents the power consumption at the sampling rate of $f_{s}$ after 24 h. When the phone is in idle state, $f_{s}$ is set as 0.

**Figure 14:**
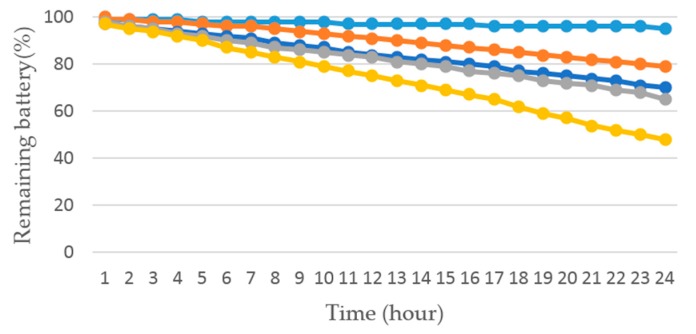
Remaining battery at different sampling rates and in idle state.

Figure 14 shows the tendency of power consumption at different sampling rates and the idle state. From the chart, we found that our ARS at the sampling rate of 1 Hz consumed 21% energy in 24 h. The ARS at the sampling rate of 5 Hz consumed 30% battery. The ARS at the sampling rate of 10 Hz and 50 Hz consumed 35% and 52% power, respectively. The battery was expended 5% when the phone was in an idle state. It can be concluded that the lower sampling rate is, the less power the phone consumed.

Table 6 summarizes the PCR in different experiment conditions. From the results presented in Figure 11 and Table 6, we can conclude that the proposed ARS of using the sampling rate of 1 Hz can save 17.3% power than the rate of 5 Hz, and there is no significant difference of accuracy achieved in the activity recognition. Comparing the sampling rates of 5 Hz or 10 Hz or 50 Hz, it can be concluded that the power consumption becomes higher with the increase of the sampling rate, but there is little improvement of accuracy. In particular, when the sampling rate increases from 10 Hz to 50 Hz, the power consumption increases 32.7%, but the accuracy only increases 0.03%. Furthermore, the ARS by using the sampling rate of 1 Hz can save 59.6% power than ARS by using the sampling rate of 50 Hz. The working time of ARS by using the sampling rate of 1 Hz is almost twice more than that of using 50 Hz.

## 5. Conclusions

This work presents a user-independent and energy-efficient ARS with high accuracy using the sampling rate lower than what is required by the Nyquist theorem. It can achieve an average accuracy of 96.0% for the recognition of six activities (sitting, standing, walking, running, climbing upstairs, and going downstairs) using the low sampling rate of 1 Hz. We also theoretically analyze the using of low sampling rate, which disagrees with the Nyquist theorem. We conclude that using a large sampling time window and a low sampling rate (such as 1 Hz) can obtain the same signal statistical properties as using a high sampling rate that meets the requirements of the Nyquist theorem.

Using a low sampling rate can save power consumption. It not only reduces the power consumption of the sensor running, but also reduces the power consumption of data processing and activity recognition. Our research shows that the ARS can save 17.3% power when using the sampling rate of 1 Hz than that of using 5 Hz, and it can save 59.6% power than that of using 50 Hz. The research has great significance for practical applications.

The H-SVM and context-based classification (HSVMCC) was proposed in our research for activity recognition. The H-SVM is an improvement of SVM algorithm, which is more efficient and achieves higher accuracy. The context-based classification is a method that uses the previous recognition results and the following recognition results to correct the current recognition results. With the integration of context-based classification presented in the paper, the average accuracy has increased by 5.1%. In comparison to the one against many multi-class approach proposed by Mortazavi et al. [31] for the activity recognition, our approach is more energy effective. It needs *N* *× (N−1)* SVM node classifiers for the *N* class classification in the one against the many multi-class approach. The HSVMCC approach only requires *(N−1)* SVM node classifiers for *N* class classification, and, therefore, consumes less power. The use of SVM with nonlinear kernel in Mortazavi et al. [31] also requires more computational cost in comparison to the proposed HSVMCC. Additionally, feature selections prior to the training and testing in our approach can greatly reduce the dimensions of the H-SVM and thereby further reduce the energy consumption.

In this paper, we also discussed the power consumption of online ARS in detail. We found that the most power consumption in the online ARS is the data processing, and the power consumption of sensor running is in a second-largest place. The power consumption used by recognition algorithms is relatively low and has very little impact on the power consumption of the entire system.

In this study, we have not yet explored whether the position of the phone can be placed arbitrarily or not, which is an important question to be further investigated in the future.

## Figures and Tables

**Table 1:** Data collection of four experiments.

Data Type	Number of Volunteers	Sampling Rate	Time Window	Overlap Time
Data collection 1	1 (male, 23 years old)	1 Hz	5 s	0 s
Data collection 2	20 (14 males, 6 females, 22–25 years old)	1 Hz	5 s	0 s
Data collection 3	5	5 Hz	1 s	0 s
Data collection 4	5	10 Hz/50 Hz	1 s	0 s

**Table 2:** Testing datasets collected from 20 participants (Data collection 2).

Activity Type	Phone Model	Number of Data Time Windows
Sitting	LG Nexus5	1234
Standing	LG Nexus5	1246
Walking	LG Nexus5	1375
Running	LG Nexus5	1154
Upstairs	LG Nexus5	1324
Downstairs	LG Nexus5	1256

**Table 3:** The performance of the H-SVM classification.

Activity	Accuracy
Sitting	97.6%
Standing	94.5%
Walking	91.1%
Running	89.1%
Upstairs	83.8%
Downstairs	89.7%
Average accuracy	90.9%

**Table 4:** The performance of H-SVM and Context-based classification (HSVMCC).

Activity	Accuracy
Sitting	99.4%
Standing	97.6%
Walking	96.6%
Running	93.8%
Upstairs	92.1%
Downstairs	96.3%
Average accuracy	96.0%

**Table 5:** Experiment setting of each case of study.

Placing State	Other Experiment Settings
Static	Running the whole ARS
Static	Only running Sensors
Static	Running the ARS without activity recognition and result processing
Static	Running the ARS without result processing
Static	The phone on standby
Shaker	Running the whole ARS
Shaker	Only running Sensors
Shaker	Running the ARS without activity recognition and result processing
Shaker	Running the ARS without result processing
Shaker	The phone on standby

**Table 6:** PCR in different experiment conditions.

Experiment Conditions	PCR
Phone in idle state	9.6%
HSVMCC by using a sampling rate of 1 Hz	40.4%
HSVMCC by using a sampling rate of 5 Hz	57.7%
HSVMCC by using a sampling rate of 10 Hz	67.3%
HSVMCC by using a sampling rate of 50 Hz	100%
